
JOURNAL INFORMATION
==============================
NLM Title Abbreviation: Behav Neurol
Journal ID: Behav Neurol
NLM Journal ID: 8914585
Journal ID: BN

Behavioural Neurology

ISSN: 0953-4180
EISSN: 1875-8584
Publisher: Wiley

ARTICLE INFORMATION
==============================
PMCID: PMC5434393
PMID: 21422544
DOI: 10.3233/BEN-2010-0284
Article ID: 352490
Article version: 1
Subjects: Research Article

Voice Recognition in Dementia

Hailstone Julia C. *
Crutch Sebastian J.
Warren Jason D.
Dementia Research Centre UCL Institute of Neurology Queen Square London UK
*Julia C. Hailstone: hailstone@drc.ion.ucl.ac.uk
Print publication date: 2010
Electronic publication date: 2011 Mar 7
Volume: 23
Issue: 4
First page: 163
Last page: 164
Received 2011 Jan 31; Accepted 2011 Jan 31
Copyright: Copyright © 2010 Hindawi Publishing Corporation and the authors.
Copyright year: 2010
License: This is an open access article distributed under the Creative Commons Attribution License, which permits unrestricted use, distribution, and reproduction in any medium, provided the original work is properly cited.
License URL: https://creativecommons.org/licenses/by/3.0/

==============================
Behavioural Neurology 23 (2010) 163–164
DOI 10.3233/BEN-2010-0284
IOS Press

### Voice recognition in dementia

Julia C. Hailstone*, Sebastian J. Crutch and Jason D. Warren
*Dementia Research Centre, UCL Institute of Neurology, Queen Square, London, UK*

### 1. Objectives

Impaired recognition of familiar voices (phonagnosia) has been described in patients with focal brain lesions [7] and in behavioural variant frontotemporal lobar degeneration (FTLD) [5], but the mechanisms that underpin deficits are less well characterised than for defective face recognition (prosopagnosia). The syndrome of semantic dementia (SD) is characterised by progressive breakdown of semantic knowledge associated with selective atrophy of the anterior temporal lobes, and offers a useful disease model in which to study different aspects of person knowledge. SD may present with progressive prosopagnosia in association with predominant atrophy of the right temporal lobe [3, 6], and voice recognition may also become impaired in such cases [3]. However, voice recognition has seldom been assessed in detail and the cognitive relations between the processing of voices, faces and other aspects of person knowledge remain to be clarified. Emerging evidence in the normal brain suggests a framework for the organisation of voice processing that is somewhat analogous to the processing of faces [1,2]. In particular, parallels can be drawn between cases of "associative" phonagnosia and associative prosopagnosia [4,5], in which impairments of familiar voice recognition occur despite intact perception of voices, supporting a hierarchical cognitive model of voice recognition in which perceptual mechanisms precede and interact with semantic mechanisms. In this study we investigated perceptual and semantic dimensions of voice processing in patients with semantic dementia (SD) and in a comparison group of patients with typical Alzheimer's disease (AD), in relation to healthy age-matched controls. Using a novel neuropsychological battery, voice recognition was compared with face and proper name recognition in order to investigate the modality specificity of any deficit.

*Corresponding author. E-mail: hailstone@drc.ion.ucl.ac.uk.

### 2. Methods

### 2.1. Subjects

14 patients fulfilling clinical criteria for semantic dementia (SD), 22 patients fulfilling criteria for Alzheimer's disease (AD) and 35 age-matched control subjects participated. General neuropsychological functions were assessed in all patients. The AD and SD groups were matched for age, education and disease severity.

### 2.2. Experimental tests

Perceptual functions were assessed using a novel test of voice discrimination (in which the task was to detect a change in speaker within a spoken phrase) and with the Benton Facial Recognition Test.

Two semantic tasks were administered in each of the three modalities (voices, faces, proper names). In the familiarity task, 48 stimuli were presented (24 famous people, 24 not famous) and the task on each trial was a "Yes/No" familiarity decision. In the second task, the same 24 famous people were re-presented separately in each modality and the task was to identify each individual (based on specific biographical information). Behavioural data were analysed using linear regression models.

ISSN 0953-4180/10/$27.50 © 2010 – IOS Press and the authors. All rights reserved

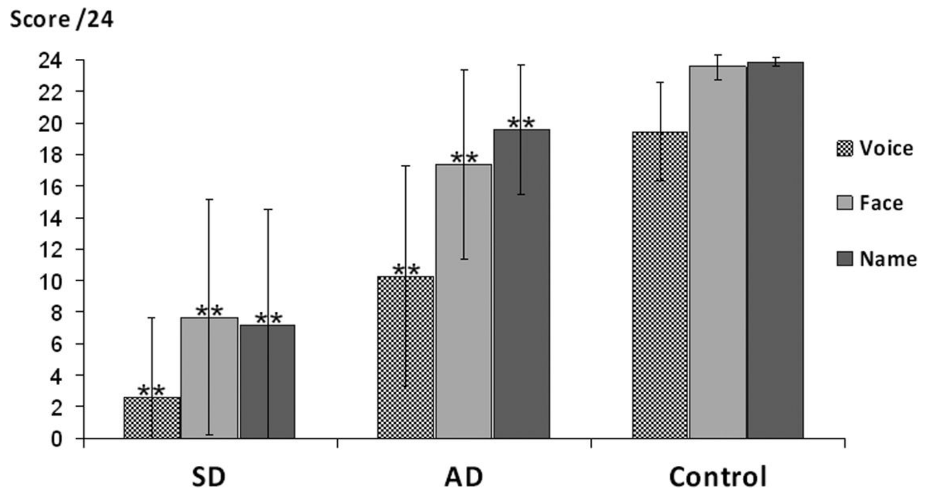

Fig. 1. Mean scores and standard deviations for voice, face and name identification tasks in SD, AD and control groups are displayed. Significant differences from controls are indicated ($p < 0.01$).

### 3. Results

The AD group showed impaired voice discrimination compared with healthy controls while the SD group performed normally on voice discrimination, however the performance of the two disease groups did not differ significantly on this task. Both disease groups showed impaired face perception performance, but again, there was no significant difference between the disease groups on this task.

Scores for identification of famous voices, faces and names are displayed in Fig. 1. A similar pattern of results across the groups was observed for familiarity and identification semantic tasks; AD and SD groups performed worse than controls at all semantic tasks, and the SD group performed significantly worse than the AD group across modalities. Relative to the AD group, processing of names was the most severely affected of the semantic modalities in the SD group.

### 4. Conclusion

Patients with common dementia syndromes have deficits of voice processing however the profile of voice deficits differs between diseases. Impaired voice recognition in SD occurs in the context of a severe multimodal deficit of person knowledge. The less severe voice recognition deficit in AD may be partially underpinned by deficient perceptual analysis.

### References

[1] P. Belin, S. Fecteau and C. Bedard, Thinking the voice: neural correlates of voice perception, *Trends in Cognitive Sciences* **8** (2004), 129–135.

[2] H.D. Ellis, D.M. Jones and N. Mosdell, Intra- and inter-modal repetition priming of familiar faces and voices, *British Journal of Psychology* **88** (1997), 143–156.

[3] G. Gainotti, M. Ferraccioli, D. Quaranta and C. Marra, Cross-modal recognition disorders for persons and other unique entities in a patient with right fronto-temporal degeneration, *Cortex* **44** (2008), 238–248.

[4] L. Garrido, F. Eisner, C. McGettigan, L. Stewart, D. Sauter, J.R. Hanley et al., Developmental phonagnosia: A selective deficit of vocal identity recognition, *Neuropsychologia* **47** (2009), 123–131.

[5] J.C. Hailstone, S.J. Crutch, M.D. Vestergaard, R.D. Patterson and J.D. Warren, Progressive associative phonagnosia: a neuropsychological analysis, *Neuropsychologia* **48** (2010), 1104–1114.

[6] K.A. Josephs, J.L. Whitwell, P. Vemuri, M.L. Senjem, B.F. Boeve, D.S. Knopman et al., The anatomic correlate of prosopagnosia in semantic dementia, *Neurology* **71** (2008), 1628–1633.

[7] D.R. Van Lancker and G.J. Canter, Impairment of voice and face recognition in patients with hemispheric damage, *Brain and Cognition* **1** (1982), 185–195.
